# Toward Sustainable Professional Development: An Investigation of Informal Interactions Among Chinese Mathematics Teachers

**DOI:** 10.3389/fpsyg.2021.681774

**Published:** 2021-07-30

**Authors:** Shu Zhang, Wenjun Zhao, Yiming Cao

**Affiliations:** ^1^College of Education for the Future, Beijing Normal University, Zhuhai, China; ^2^Center for Mathematics and Mathematical Education, Beijing Normal University, Zhuhai, China; ^3^School of Mathematical Sciences, Beijing Normal University, Beijing, China; ^4^International Center for Research in Mathematics Education, Beijing Normal University, Beijing, China

**Keywords:** informal interaction, community of practice, sustainable professional development, teacher reflection, Chinese mathematics teachers

## Abstract

It is difficult for teachers to achieve sustainable professional development without support from other teachers. Many researchers have stated that teachers regard informal interactions in daily practice as crucial to learning from one another. In this paper, we present a study in which we investigated informal interactions between Chinese middle school mathematics teachers. Three dimensions of their interactions were identified through an analysis of semi-structured interviews. The data revealed how teachers initiate informal interactions based on shared goals, how they meet expectations of the dual roles of teaching and educational research, and how they perceive the effects of informal interactions on their teaching practices. These results contribute to a better understanding of the process of informal interactions from individual teacher perspectives. In this paper, we also discuss further implications for enhancing sustainable professional teacher development with daily practices.

## Introduction

It has been difficult to bring innovation to the practicalities of teaching, especially across different educational systems. Research has shown that collaboration among mathematics teachers plays a critical role in supporting their professional development as they work to improve teaching practices (Borko, 2004). For example, the recent 25th International Commission on Mathematical Instruction (ICMI) study group paid particular attention to how mathematics teachers work in different situations and how collaboration might lead to improvements in expertise and practice. Numerous studies have focused on formal collaborative programs, and the results from this research show that teacher learning may occur within the context of organized professional development activities (Bell et al., 2010; Huang and Shimizu, 2016). However, the sustainability of teacher learning in such activities has been noted as an issue in mathematics educational research, partly because of the inconsistency in formal professional development activities (Robutti et al., 2016; Jaworski et al., 2017). Informal interactions are also regarded as a crucial part of teacher collaboration and are essential to sustaining the process of teacher learning.

To clarify the terms, “informal interactions” refer to reciprocal verbal communications and actions among teachers that are not officially organized by any parties. That is, we particularly focused on interactions that were self-initiated and happened spontaneously without any specific organization or particular form. The words “communication,” “conversation,” “discussion,” and “talk” all refer to such interactions. Therefore, we used these words interchangeably to denote “interactions.”

Numerous study results have shown that informal interactions with one another contribute to joint work among teachers. Informal interactions contribute to collaborations, which provide opportunities for teachers to learn and improve their skills (Coutinho and Lisbôa, 2013; Brodie, 2020). Over the past two decades, a trend to emphasize the importance of a collaborative element in teacher professional development has been noted (Youngs and King, 2002). To improve the outcome of teacher professional development, it has been argued that teacher interactions should be conducted within the school context (Park and Lee, 2015). Yet, because of the various forms and the flexibility of informal interactions in different educational systems, little has been reported on the practice among mathematics teachers.

In the Chinese collective culture, teachers have traditionally worked in groups. A variety of groups exist at schools, and normally, groups have their own offices, which makes informal interactions more accessible (Chen and Yang, 2013). Research has shown that expertise among Chinese teachers is improved through a variety of organized teaching research group activities, which also contribute to building a common space for informal interactions at school, as they share common experiences during these group activities (Chen and Yang, 2013; Wei, 2019). However, little is known about how informal interactions among the various groups constitute this important part of their professional development.

In this study, we aimed to investigate how Chinese mathematics teachers conduct informal interactions; that is, we wanted to explore the “what,” “how,” and “why” of their informal interactions. An understanding of the contextual characteristics of informal interactions between teachers within Chinese schools may provide possible implications for sustainable professional development in other contexts.

## Literature Review

### Research Background

In Chinese schools, professional development for teachers was intended to be guaranteed through three levels of organization: national, district, and school. As with most countries, a teacher's qualifying education and ongoing in-service training are used to ensure rigorous levels of professionalism. This is achieved at the national level. At the district level, a local educational research institution is used to provide resources for professional development, including, but not limited to, school collaboration in various forms and collective participation in local lectures. Alongside this training, a school-level professional development system is important for teachers. As noted by Chen and Yang (2013), the school-based professional development of mathematics teachers is traditionally and culturally rooted in the school system because of China's collective culture. Cao and Li (2018) introduced the Chinese school-based professional development system in detail. In their paper, the school-based professional development system is shown to include group activities, such as research, lesson planning, observation and assessment of classrooms, and master–apprentice tutoring for new teachers. Outside of group activities, daily consultations with school colleagues are also valuable professional development tools.

For in-service training, the teaching research group (TRG) and the lesson planning group (LPG) each play critical roles in fostering school-based professional development activities in Chinese schools. According to the results of a large-scale survey of Chinese mathematics teachers, there are different types of teaching research activities in the TRG at each school, including public lectures for enhancing theoretical learning, periodical work arrangements and summaries, public class assessments, and organizing workshops that focus on the practice of teaching and issues with it. Overall, the aims of TRG activities are to provide macro-guidance for solving daily problems and creating opportunities for idea exchanges with teachers of different grades (Cao and Li, 2018).

The LPG is small-scale, as it is usually composed of mathematics teachers who teach the same grades. The main goal of this group, inherent in its name, is for members to meet regularly and plan lessons together. China has one uniform curriculum standard for all schools, which means that student academic assessments are based on learning curriculum-standard knowledge. In some schools, teachers must maintain the same strategy for each topic; in other schools, they can use different approaches while essentially discussing the main concepts. They also discuss how to help students understand mathematical concepts and ideas, what is worthy of more attention and should be focused on, and how to bolster student learning (Cao and Li, 2018). In contrast to the TRGs, the LPGs aim to provide micro-level guidance for developing lessons with regard to understanding the topic, designing teaching sections, introducing prior knowledge, and selecting examples and exercises. It is also worth noting that each group can be different in each school, and that depends on how schools organize teaching research and lesson planning activities. It is possible that some teachers belong to both groups, while others belong to one group, and yet others belong to neither group.

In comparison with formal group activities, informal daily interactions between teachers are also an important source of professional development. Data from a quantitative study of mathematics teachers from three cities in China show that teachers tend to carry out informal interactions with their peers in research and lesson planning groups (Cao et al., 2020). When encountering instructional problems, colleagues from these groups usually provided the most direct suggestions and assistance. Their group leaders showed notable initiative and played a leading role in solving problems and answering questions (Cao et al., 2020).

It cannot be denied that organized, school-based professional development activities provide resources and opportunities for teachers to learn both theoretically and practically. It could be argued that these activities also create an environment and a foundation for teachers to engage in further self-initiated interactions with their colleagues. However, the mechanism by which this happens is unclear; in this study, we aimed to address this gap.

### Research on Teachers' Informal Interactions

The significance of teacher interactions relevant to professional development has been widely researched. However, scholars have approached the topic from a variety of perspectives.

With the popularity of lesson study (Chen and Yang, 2013; Lewis and Lee, 2017), researchers have gradually become more interested in how different groups of people involved in the educational system interact with each other and how these interactions may influence teaching and learning (e.g., Shirrell et al., 2019). Robutti et al. (2016) stressed that interactions among different groups of people in mathematics education might increase the quality of teaching. However, teacher interactions include those that happen during organized workshops, discussions, and lectures, as well as conversations that are self-initiated during daily practice, after lessons are taught, during break times, and otherwise outside the classroom. A focus on daily teacher interactions and involvement with colleagues has also been addressed in the literature (Penuel et al., 2012).

At the individual level, teachers might be more willing to casually interact with their peers in daily practice than in formal professional development activities. This is because casual interactions are free from routine principles, which means that these informal interactions are more self-driven and situated in real issues at the moment when teachers need help. From the perspective of emotional responses, Hargreaves (2001) interviewed 53 Canadian teachers and found that they valued the personal support and acceptance they received from their colleagues.

When it comes to interactive activities, informal interactions usually involve teachers with various responsibilities, including headmaster, leader, or classroom teacher. The multiple roles that teachers might play during interactions have become a focus for certain researchers. Some are interested in investigating how specific roles may vary in different contexts. For example, in lesson studies, the role of the “knowledgeable other” can vary within and across cultural contexts (Adler and Alshwaikh, 2019). While some researchers concern themselves with the various roles that one teacher might play in the same context, Widjaja and Vale (2020) paid attention to the dual roles that a lead teacher might play as a coach or curriculum coordinator, as well as a member of a lesson planning team. Rather than focusing on the various roles that one teacher might play, Qian and Walker (2020) examined how Chinese school leaders conduct their roles as leaders to create the structural, cultural, and relational conditions in which teachers conduct collaborative learning activities during daily practice. These studies refer to informal relationships between teachers and colleagues.

Informal teacher interactions have also been studied from the perspective of how they might influence instructional changes in practice. Penuel et al. (2009) argued that teacher interactions play a positive role and allow for the exchange of resources and points of expertise in practice. Penuel et al. (2012) noted that exposure to interactions with colleagues may allow for the prediction of changes in instructional practices. For those who engage in practice more frequently, this change may be even stronger than is realized when directly participating in organized professional development activities. This means that in situations when formal activities are difficult to organize, informal interactions between teachers may be a critical tool for their professional development in daily practice. However, as noted by the authors of both studies, their research was designed from a quantitative paradigm; a qualitative analysis of interactional content is needed to further investigate the process by which interactions influence pedagogical practices.

In the context of educational reform, researchers have noted that informal interactions between teachers can also help achieve sustainable implementation. This is because they may find it easier to understand new principles in their own language when a theory is novel to them. For example, Chen and Yang (2013) provided insights into the construction of a reform-based teaching strategy. They concluded that those within the school context have a shared interpretation system that may be revealed by their “native discourse” (consisting of teachers' daily language use, concepts, and interactions). Zhao et al. (2020) studied reform-based teaching practices and concluded that an atmosphere and place for interactions helped mathematics teachers put reform theories into practice. This study showed that informal interactions allow people to connect with the specific advantage of an easy understanding of language.

Additionally, in most studies on teacher interactions, researchers seem concerned about the role that interactions play in providing teachers with hands-on experiences. Penuel et al. (2012) suggested that, through informal interactions, they may give and receive help to draw on experiences and points of expertise developed from the situations at hand. Similarly, Heck et al. (2019) found that collective discussions were valued by mathematics teachers, echoing Fishman's et al. (2013) question about whether interactions with colleagues might allow teachers to draw from others' hands-on experiences. The ability to create various opportunities for sharing hands-on experiences reflects the flexibility of informal interactions, which allows teachers to promptly translate information into practice.

Regarding daily interactions, Cross et al. (2002) noted that some significant outcomes of teacher learning occur in daily conversations, during which teachers exchange their ideas and opinions about the field. These informal talks promote knowledge development and understanding, display creativity in teaching, and encourage changes in the classroom. Sánchez (2011) also reported results from an investigation of interactions based on an Internet-based mathematics education program. The data showed that teachers were involved in meaningful interactions when they could “express, justify, compare, and evaluate their own mathematical and pedagogical ideas” (Sánchez, 2011, p. 106). These interactions allowed the teachers to reflect on their own methods, drawing from the similarities and differences they found when comparing their methods to those of other teachers.

Although the significance of informal interactions among teachers has been addressed in the literature, the specific process of informal interactions, in terms of how teachers initiate and sustain them, what occurs, and how they view the influence of these interactions, is still not clear (Siu, 2015). In this paper, we agree with the assertion that informal interactions may play a critical role in sustaining professional development if used well. In China, school-based professional development activities are widely available throughout the country (Huang et al., 2017, 2018). Under such a context, we aimed to investigate how informal interactions were implemented in daily practice among teachers.

### Theoretical Perspectives

Discussions about teachers' informal interactions in a learning community have mostly been derived from the framework of the community of practice (CoP) developed by Etienne Wenger (1998). A “community of practice” refers to “groups of people who share a concern, a set of problems, or a passion about a topic, and who deepen their knowledge and expertise in this area by interacting on an ongoing basis” (Wenger et al., 2002, p. 4). An obvious characteristic of a CoP is its self-organizational nature of people with common interests. The group is designed to deliver and exchange knowledge and expertise as a shared practice among interested members (Wenger, 1998; Wenger et al., 2002). Communities of practice can be either formal in an organization or informal (Wenger, 1998). Based on situated learning theory, adults are able to socialize their knowledge through interactions in the CoP (Lave and Wenger, 1991; Smith and Sadler-Smith, 2006). Evidence from previous research shows that informal interactions can be analyzed from the perspective of a teacher's CoP (Seaman, 2008; Siu, 2015; Van Lankveld et al., 2016). In this community, teachers are able to share their “expertise, competences, learning activities, discussions, information, tools, stories, experiences, and a knowledge base” (Seaman, 2008, p. 270); they may negotiate meaning, form common goals, and build a teaching identity to help them collaborate and learn (e.g., Goos and Bennison, 2008). Therefore, in this paper, we followed the lead of other researchers and chose the CoP as our conceptual framework.

**Figure 1:**
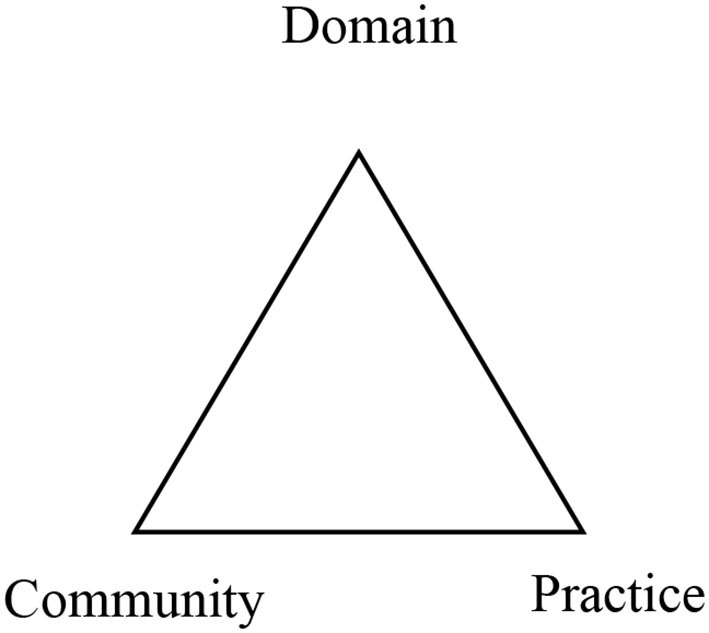
The community of practice framework.

Although other researchers have tried to recognize the key characteristics of a CoP from different perspectives (e.g., Cobb et al., 2003; Pearce, 2010; Van Lankveld et al., 2016), there are clear commonalities and similarities regarding the essentials of a CoP. As opposed to other communities where member responsibilities and duties are clearly stated, and there might be certain rules restricting members from forming communities, informal teacher interactions are usually flexible and self-initiated, which makes these communities rather weak in terms of organization. Wenger et al. (2002) explained that when a CoP is relatively weak, communities can be created by paying attention to the following three essential dimensions (Figure 1):

- Domain—what the community is about, as well as the shared knowledge, goals, and purpose of the community.
- Community—the relationships between members, and how members interact with each other.
- Practice—sharing understanding with other members of the community, which develops and maintains knowledge and skills in the shared Domain.

In this paper, we draw on the three constituent dimensions of the CoP to operationally analyze informal interactions between teachers (Wenger, 1998). The shared Domain in the current context describes the shared space, problems, goals, and beliefs among teachers in daily informal interactions. Community represents the relationships between teachers in their respective roles, while Practice refers to the perceived effects and influences of informal interactions on teachers' daily practices. In existing literature, although researchers have revealed that informal interactions demonstrate some characteristics of a CoP (Siu, 2015), the details of how teachers behave in each dimension, and how the different dimensions relate to each other in terms of affecting teacher learning, still remain unclear. Applying the lens of a CoP to the context of Chinese mathematics teachers provides us with a window to observe these details.

### Purpose of This Study

The purpose of this study was to examine how Chinese mathematics teachers participate in informal interactions as part of their daily teaching practice, using the framework of a CoP. With regard to theoretical perspectives, this study was designed to address the following research questions:

1. What brings mathematics teachers together to have informal interactions (Domain)?
2. What are the relationships among teachers and their roles in informal interactions (Community)?
3. What are the perceived effects that teachers seek through informal interactions (Practice)?

## Methods

### Data Collection

The data reported in this paper were drawn from the Chinese government-supported project Alignment between Theory and Practice of Chinese Mathematics Curriculum Reform. As part of the project, informal interactions between mathematics teachers were investigated in two schools. We chose these particular schools based on an initial investigation of informal interactions between teachers there (Cao et al., 2020); we found that mathematics teachers in these two schools were willing to share details about their informal interactions, and they valued the significance of informal interactions in their teaching practices. Compared with teachers from other schools we approached during this project, the teachers at these two schools reported more information about informal interactions. The two groups of teachers consisted of four mathematics teachers from School A and five from School B in the cities BJ and CQ (see Table 1). Both were key schools in their respective local districts. In China, key schools are those in which students achieve relatively higher grades than others in the same district or city. Individual, semi-structured interviews were conducted with each teacher after the completion of a questionnaire. Each interview lasted 40–60 mins and took place at the local school. The following main interview questions were posed to guide the interview:

1. How often do you have informal interactions with colleagues at school?
2. What brings you to informal interactions with colleagues? What topics do you usually discuss when having informal interactions?
3. With whom do you normally engage in informal interactions, and why?
4. How do you think informal interactions with colleagues influence your professional development?

**Table 1:** Teachers' demographic information.

**Teacher**	**School**	**Teaching grade**	**Teaching experience**	**City**	**Groups (TRG/LPG)**
A1	A	7	16	BJ	TRG, LPG
A2	A	7	15	BJ	TRG, LPG
A3	A	7	8	BJ	TRG, LPG
A4	A	7	17	BJ	TRG, LPG
B1	B	7	17	CQ	TRG, LPG
B2	B	7	3	CQ	TRG, LPG
B3	B	7	9	CQ	LPG
B4	B	7	17	CQ	TRG, LPG
B5	B	7	4	CQ	LPG

### Data Analysis

Data analysis was performed from an interpretivist point of view because it fits with our aim of constructing insightful accounts of interactions (Geertz, 1983). First, we selected useful information from the teachers' comments related to informal interactions. Then we combined the selected information with the observation notes and summarized how teachers in each school conducted informal interactions. A general characteristic of each school was determined—for example, how teachers communicated with each other and whether teachers showed positive attitudes and emotions during interactions. This gave us a basic understanding of the background and a general picture of the frequency of informal interactions, as well as which teachers interacted.

To examine the process of these interactions, the interview data were analyzed by applying a thematic analysis (Braun and Clarke, 2013). To capture the themes and codes that could be used to describe informal interactions in the interviews, a constant comparison coding process was conducted (Strauss and Corbin, 1998), during which the researchers read the texts and highlighted related information about informal interactions based on the literature and researcher understanding of each school's context. Then we open-coded specific parts of the content and incidents that seemed critical and common, as mentioned by teachers. The next step was to construct thematic categories based on connections identified among the codes in the open-coding process and align them with the implemented theoretical framework. Three researchers were assigned to conduct the coding process independently from December 2019 to March 2020. Later discussion was organized to resolve any disagreement until all researchers achieved alignment. The research team repeatedly reviewed the interview transcripts to ensure that the interpretations aligned with teacher descriptions. The final unit of analysis, in terms of detailed descriptions of the theme categories, was validated through triangulation via discussion among members of the research team. It is also worth noting that to avoid any ambiguity in coding, we restricted our coding of texts under each interview question we asked, i.e., the themes under the Domain category were all derived from teacher feedback about the first interview question when we asked about the Domain. The resulting coding themes and categories are presented in Table 2.

**Table 2:** Coding themes and categories.

**Domain**	**Community**	**Practice**
• A shared goal of understanding the curriculum standard	• The role of group leaders as institutional organizers and facilitators	• Gain a better understanding of teaching requirements and standards
• A shared goal of improving teaching	• The role of group leaders as expert teachers	• Develop teaching skills and strategies
• The need for hands-on experience		• Reflect on teaching effectiveness
		• Broaden new perspectives on instructions

## Results

### An Overview of Teachers' Informal Interactions at Schools

At different schools, teachers may have preferences about whether they wish to engage in informal interactions. Unlike formal professional development activities, informal interactions happen casually and flexibly among teachers. Based on the results from the interviews with the nine participants, descriptions of the informal interactions in terms of frequency and participants is summarized in Table 3 (see also Guo, 2012). It is obvious from this data that most teachers have informal conversations with colleagues in daily practice, especially when encountering difficulties and problems.

**Table 3:** Frequency and participation in informal interactions.

**Teachers**	**Frequency of informal interactions**	**Participants in informal interactions**
A1	2–3 times per day	TRG leader, colleagues in the LPG (A2, A3, AL and so on)
A2	When having problems and questions	A1, TRG leader, or LPG leader
A3	Once a week with A1, sometimes with others, and the TRG leader	A1, TRG leader
A4	Once per day after the lesson is taught	A1, A3, AL^*^, colleagues in LPG
B1	Once every 2 weeks	Colleagues in LPG, LPG leader
B2	Sometimes	Colleagues in LPG, LPG leader
B3	Four to five times per day	Colleagues in LPG, LPG leader
B4	Not much	Colleagues at the same grade
B5	Once every 2 days	Colleagues, LPG leader, teaching research specialist

* *AL belongs to the LPG of school A but did not participate in the study. A teacher research specialist was usually in charge of the teaching research activities of a district, which includes all the schools of that district*.

For the four teachers at School A, it is worth mentioning that at the time of the interviews, participants A1, A2, and A3 had been working at the school for more than 8 years, while participant A4 had been working there for 1 year. Participant A1 reported the most frequent interactions among the four teachers, as his informal interactions happened 2–3 times per day with the teaching research team leader or colleagues within the same LPG. Participant A2 stated that she asked participant A1 questions when she had problems or points of confusion. Participant A3 said she might have conversations with Participant A1 and the teaching research team leader, usually with Participant A1 alone once a week, and casually with other colleagues. Participant A4 tended to talk with participant A1 about problems with teaching or students after each lesson.

For the five teachers at School B, all had been working at the same school since they started teaching. Participant B1 had informal interactions with colleagues or the LPG leader approximately once every 2 weeks to “maintain her teaching align[ment] with the whole group's plan” (B1). Participant B2 said that she sometimes had informal interactions with colleagues or the TRG leader when she had concerns and questions about methods and practices. Participant B3 had informal interactions with colleagues and LPG leaders every day because she liked to “understand clearly about the teaching requirements and share students' problems and learn teaching skills from others” (B3). Participant B4 rarely had informal interactions with others. Participant B5 said she had informal interactions with other teachers, such as colleagues and LPG leaders, usually once every 2 days. She said she might also communicate with the teaching research specialist when that person came to their school and participated in LPG activities.

As observed in the interviews, most teachers have informal interactions with other teachers. Among the interactive participants, other than colleagues, the TRG leader and the LPG leader were often mentioned. This shows the explicit role of group leaders in informal interactions between teachers. For the four teachers at School A, three teachers (participants A2, A3, and A4) all referred to Participant A1 as a subject for informal interactions. Participant A1 also mentioned the other three teachers in her descriptions. This, to some extent, reveals the mutuality of the relationships among the four teachers. In contrast to those from School A, the five teachers at School B tended not to mention the specific person with whom they interacted, with the exception that they still mentioned the LPG leader. The TRG leader was not mentioned very often. This seems to indicate that the LPG leader played a more active role in teachers' informal interactions at School B.

### What Brings Teachers Together for Informal Interactions (Domain)?

Three themes emerged regarding shared goals and problems; together, these formulated the Domain for informal interactions between teachers and helped initiate interactions.

#### The Shared Goal of Understanding the Curriculum Standard

According to the participants, learning the curriculum standard has always been the focus of professional development activities. The school-based TRG and LPG activities all assume the “learning of curriculum principles and standards” as the main goal. Teachers must know what content the curriculum standard documents cover, and they must understand how the principles from that standard can be put into practice. However, learning the curriculum was reported as usually conducted through public lectures of content introduction, which may not fit well with the practical aspects of teaching specific content. This makes a deep understanding of curriculum principles and the standard a shared first goal and initiation point for informal interactions, as participants A4 and B3 stated:

A4: “I think it's helpful when we invite teaching research specialists and other experts, like, you know, researchers, to the school and organize public lectures or workshops, because the experts usually may mention a macro-level curriculum standard, for example, like what are the new reform-based teaching ideas proposed by the curriculum standard, or maybe other countries' teaching principles, etc. We might all like the ideas, so we would informally discuss the ideas and principles after the public lectures and see if we could put the curriculum standard into practice or to what extent we could draw from the new ideas in our schools.”

B3: “… some teachers have more knowledge and deeper understanding toward the curriculum standard than their colleagues. For example, the curriculum standard stated that we ‘need to help students to improve their number sense,' then how to enact ‘number sense' into classroom teaching. Some expert teachers could probably give more explanations for that.”

#### The Shared Goal of Improving Teaching

Along with understanding the curriculum standard, teachers shared the goal of improving teaching, which also helped initiate informal interactions. From the analysis of the teachers' interviews, this goal has two aspects: (1) learning content knowledge of mathematics and (2) developing teaching skills and improving knowledge about their students.

For this first aspect of learning content, schools that teach mathematics to Year 7 students may organize practices based on the exams so students can be assessed together. Teachers in the same office may also have their classes perform the practice exercises separately and then communicate with each other during breaks about the practice exams, including what knowledge was assessed and how it was assessed. Participants A3 and B1 both mentioned how mathematical knowledge might be shared in informal interactions:

A3: “Our teachers will complete the exam tests sometimes together, sometimes on our own; for example, some practices from the past entrance exams for high schools. We then would talk about the practices because I think doing practices is also part of learning… for teachers. We need to talk about how the problem could assess students' knowledge in like some mathematical ideas, etc.”

B1: “… we can discuss the content knowledge, practices, and concepts that students may find difficult in the office. We learn from other teachers about how they understand the concept…”

The second aspect of improving teaching is developing instructional skills and learning more about students. Participants A1, B4, and B5 talked about the need for communicating how to improve teaching skills, such as designing lessons, pedagogical theories, and different teaching strategies:

A1: “When talking with colleagues from the same lesson planning group, we usually share each other's ideas in designing the lesson, observing the lesson, and teaching feelings and experiences after the lesson; this allows [us] to accumulate experiences for further teaching.”

B4: “Sometimes, it is good to discuss pedagogical problems with colleagues; for example, for some units, it is good to teach through only teacher lecturing, while for some units, we may want to enrich students' learning experiences, like organiz[ing] collaborative problem-solving activities. It is good to know how other teachers might organize their teaching sections during one lesson.”

B5: “We might discuss students' difficulties in past teaching; based on this, we can then propose what we need to pay attention [to] in further teaching.”

These remarks suggest that the shared goal of improving teaching allows teachers to initiate informal interactions about how to design lessons, how to solve difficulties and problems in teaching practices, and how to reflect on their own teaching.

#### The Need for Sharing Hands-On Experiences

Sharing hands-on experiences can also be seen as an important initiation for informal interactions. Participants said they thought teaching could involve a huge onset of problems at any time. To solve these problems in real time, informal interactions are essential because schools cannot organize formal workshops to discuss all the problems that occur in daily teaching. Especially for new teachers, hands-on experiences within the context of the school can be useful for development. Participants A4, B2, and A2 all mentioned that they needed these exchanges in daily teaching:

A4: “I still remembered when I first came to the school, I was not familiar with students' backgrounds and also the schools' preferences in terms of teaching styles or office culture, etc. It takes time to get along with students and colleagues when you come to a new school, but casual talks and break discussions helped a lot in providing advice for a lot of issues, like, even classroom management, etc.”

B2: “I actually want to know how other teachers solve students' questions and problems, because students in their classrooms may have similar problems with mine, then I can avoid [these problems] in advance upon teaching, or I can draw from their experiences.”

A2: “There could be a lot of tiny problems in daily mathematics teaching. For example, students may have misconceptions for some mathematical concepts, but we may not know how to solve these problems. But when you share with other colleagues, they could probably come up with good ideas based on their experiences, and it won't take too much time either.”

For the domain of informal interactions between teachers, shared goals of understanding the curriculum standard, improving teaching, and sharing hands-on experiences can initiate informal interactions among teachers. These shared goals allow teachers to have a common topic to discuss, which may strengthen their interpersonal relationships.

### How and With Whom Teachers Informally Interact in Daily Practice

As stated in An Overview of Teachers' Informal Interactions at Schools section, although most participants mentioned their interactions with colleagues at schools, another commonality was that almost all teachers specifically referred to their interactions with either the teaching research specialist or the LPG leader, and their interactions with these group leaders were usually more frequent than with other colleagues. Two themes about how these group leaders functioned in their dual roles during informal interactions were evident from the analysis. In informal interactions, teachers may play different roles; for example, they may function as either a knowledgeable person or a person seeking help. The dual roles identified in this study mean that lead teachers are not only institutional organizers and facilitators, but also experts.

#### The Role of the Group Leader as Institutional Organizer and Facilitator

Team leaders take responsibility for organizing TRG and LPG activities. For the TRG, responsibility usually includes setting a long-term teaching plan for each grade or organizing a group meeting for lesson preparation, teaching plan design, or control of teaching stage. More specifically, the overall meeting topics include the content, depth, sequence, and pace needed to stay at the same level as other teachers in the same grade across the nation.

For the LPG, the leader plays the role of organizer. The basic group activity is preparing lessons together (including adjustments to the teaching stage, standards, and aims), organizing group teaching plans, and observing lessons. Participants A2 and A3 stated why they must have informal interactions with the TRG leader and/or the LPG leader related to macro-level control of teaching:

A2: “For teaching, the lesson planning group leader takes the responsibility to control the teaching pace, difficulty, and other aspects of teaching activity. (I) need to know the overall teaching goal of the whole lesson planning perspective.”

A3: “The group leader controls the whole group's or the whole grade's teaching standard. If I only talk with other teachers rather than AX [name of the group leader], we may not have a full understanding of the whole group's goal.”

Therefore, teachers must have informal conversations with group leaders to ensure that each plan aligns with the overall educational arrangement.

#### The Role of the Group Leader as an Expert Teacher

Group leaders are also teachers who usually have more experience and/or professional achievements in the field. At both schools, the group leaders are teaching experts within the school or the local district. Therefore, their professional knowledge and skills are regarded as important resources for teachers, especially when faculty members have difficulties. Teachers believe that rich instructional experiences and shared stories of developing pedagogical strategies from group leaders are useful for coping with problems, such as helping students overcome misconceptions. Participants B1 and B3 spoke of the expertise provided by their lead teachers and stated why they must have informal interactions with them:

B1: “As a teaching expert, the lesson planning group leader might stand higher when it comes to knowledge content and knowledge points.”

B3: “The expert teachers have more experience in terms of coping with students' problems, like, they know when students have made mistakes, what can be done to help students understand the rooted reason of their mistakes. They also helped us to reflect on our teaching strategy so as to see if I can make any improvements.”

It is easy to acknowledge that informal interactions occur between teachers and group leaders, as well as among teachers themselves. However, in this study, we specifically addressed the dual role that group leaders play in stimulating informal interactions and helping teachers with problems. The relationship between regular teachers and group leaders may be regarded as one of the stimulus points for such activities.

### Teachers' Perceptions of the Effects of Informal Interactions

Informal interactions occur for a variety of reasons, and they relate to different aspects of teaching, including the shared curriculum standard, teaching activities, and teaching assessments. Based on the analytic framework, the third dimension of the community of informal interactions is Practice, which is how teachers might reflect on their informal interactions with each other (see Table 2). Four themes came to light in terms of the perceived effects of these interactions, as identified below.

#### Gain a Better Understanding of Teaching Requirements and the Curriculum Standard

According to the teachers, informal interactions allow them to communicate the teaching standard in detail. Through informal conversations with colleagues teaching the same subjects, they may be able to gain a more general and practical understanding of the teaching standard, such as determining the extent to which certain knowledge should be taught based on difficulty. Participants A2 and A3 suggested that informal communication with other colleagues helps them maintain their teaching under the curriculum standard:

A2: “I need to ensure that my teaching is within the teaching standard, and the knowledge I [teach] is useful for students to achieve their learning goals.”

A3: “The standard set requirements and content range for teaching, to master it; talking with colleagues [is] helpful for understanding the requirements and content range.”

Although teachers are provided with learning materials, such as the curriculum standard and reference textbooks, the language is often rather academic and, therefore, sometimes difficult for teachers to put into practice. Participant B4 stated that casual conversations among colleagues makes academic language much easier to understand:

B4: “…you know, the standard shows the requirements in a general form and [an] academic way; it aims to the students of the whole country. In practice, teaching situations vary with each other; students are also different. At the same school, students' background and prior knowledge might not differ too much; we have common language with colleagues. It is more convenient to understand the standard and requirements for student learning.”

As seen above, informal interactions allow teachers to use spoken language and even their own expressions to achieve a common understanding of the standard.

#### Developing Teaching Skills and Strategies

Informal interactions provide opportunities for teachers to elaborate on their methods and strategies. Teachers might share stories of student feedback and their interactions with students during the lesson. Participants B1 and A4 mentioned that sharing student feedback would provide other professionals with resources for improving teaching methods and understanding student learning:

B1: “Each teacher has different teaching styles; (I can) know more teaching plans through informal interactions. For example, some teachers might organize the teaching content and knowledge points clearly and logically; I need to learn how to organize the order of teaching content occurring during the lesson.”

A4: “Students have different methods for solving a problem, especially in different classrooms. Through informal conversations with other teachers, I can even learn more different problem-solving strategies from students in other classes.”

The teachers reported that specific methods for improving teaching strategies and skills were shared and communicated in informal interactions in daily teaching.

#### Reflections on Teaching Effects (on Students)

Informal interactions between teachers also include reflections on their own instructional styles, as well as student learning behaviors. In both schools, after each unit of teaching, the LPG might organize student assessments of the content knowledge learned during the whole teaching unit. After these assessments, teachers communicate with each other regarding their students' grades and discuss whether the learning goals were achieved. Based on the tests, teachers are able to know to what extent each student acquired the knowledge, and which students might need after-class tutoring. Participant B4 stated that her communication with other colleagues gave her a sense of whether students performed well (in terms of their academic performance during classroom teaching) compared to others in different classes. This allows for further adjustments to her approach:

B4: “The reason why I need to talk with other colleagues is that we need to check whether some students did not achieve the learning goals, and what did he or she know or not know about the knowledge so that we can design practices or [tutoring] for these students to help them follow the learning stages.”

#### Broadening New Perspectives

The respondents indicated that informal interactions with different teachers may also broaden their horizons as they learn new perspectives from others. Teachers might have different beliefs in terms of teaching, which can be shown from their activities, as explained by participant A1:

A1: “For my students, sometimes they submit their homework, and I find that they can complete their homework very well. I know some students may not learn that well, but if students go back home to do homework, their parents might also help on doing homework. However, my colleague explained to me that she asked parents to not help on students' homework, and to help ensure that students finish homework [on] their own, so that she can identify each student's problems instantly. I think this is a good idea because it solves the problem through the opposite side. Most teachers would ask parents to help students on homework rather than not to do so.”

The four themes partly reflected the shared Domain of teachers' informal interactions, as stated earlier. They gain a clear understanding of teaching requirements and the curriculum standard as they develop teaching methods and strategies that echo the shared goals of understanding the standard and improving teaching skills. This means that teachers can, to some extent, achieve their goals through informal interactions. This is a crucial reason for them to sustain daily informal interactions. Reflecting on their own teaching effects and broadening their perspectives are “bonuses” of informal interactions among teachers, which also drive them to continue engaging with each other.

## Discussion

As shown in Table 2, the shared Domain of informal interactions includes a common goal for understanding principles and the standard, improving teaching, and solving problems. The Community illustrates the critical role that the research group leader and LPG leader play in becoming a person others look to for guidance. The results of this study provide an understanding of how teachers perceive the effects of informal interactions in terms of the four dimensions of their teaching practices.

### Shared Goals Guide the Content of Informal Interactions

Based on knowledge of Chinese school-based professional development activities, and as revealed through teacher interviews, the shared Domain correlates with school-based, formal professional development activities, such as participation in the TRG. Since this group's activities provide theoretical learning of curriculum principles and the standard, an important shared goal of informal interactions among teachers is to enhance understanding after formal learning has occurred. Because China has one uniform curriculum standard for all schools, and student assessments are based on this standard, teachers must be transparent about its requirements and what should be taught, so consistency is maintained between the standard and their teaching (Zhang et al., 2019).

China started a new round of curriculum reform at the beginning of the twenty-first century. After years of trials and experiments to improve teaching, the country is still developing effective and innovative teaching skills. The LPG organizes activities to try out new teaching strategies and pedagogical ideas that align with the implementation of educational reform (Zhao et al., 2020). However, teachers must realize a common goal for understanding and developing skills under the new paradigm, such as teaching through collaborative mathematics problem-solving activities, to improve academic performance levels. As we presented in What Brings Teachers Together for Informal Interactions (Domain)? section, participants in this study reported that informal interactions help them to clarify technical terms from the new standard and to further reflect on how to put teaching theories into practice. Informal interactions regarding content discussed in LPG activities also allow teachers to achieve a reasonable level of understanding, thus improving their abilities.

At the same time, these professionals often share problems relating to student difficulties, along with topics such as teaching design, mathematics content knowledge, and even classroom management. In this study, the interviewees stated that the hands-on experiences shared through informal interactions were valued. For teachers, there is no one-size-fits-all recipe for teaching because teachers and students vary from classroom to classroom; not everyone is the same (Sfard, 1998). Shirrell et al. (2019) noted that reform-oriented instructional changes were driven by informal interactions rather than by formal learning activities. This study further illuminated this finding and suggested that informal interactions can be seen as a complementary learning approach beyond formal learning activities.

### The Critical Role of Group Leaders in Building an Atmosphere for Informal Interactions Among Teachers

In the Community dimension of the theoretical framework, it was identified that group leaders play a critical dual role in promoting informal interactions between teachers. As opposed to previous studies that observed CoPs that depended on bonded relations and responsibilities toward other teachers (e.g., Cobb et al., 2003), there are no such responsibilities in the CoP of informal interactions; the teachers can actually enter and quit the community very freely. However, in contrast to our expectations, our participants were all willing to be involved in informal interactions, and almost every teacher mentioned their informal interaction with group leaders. This willingness suggests that the group leaders have shaped a positive learning culture and a place where teachers can trust each other at both schools.

Previous research revealed that leaders at schools actually “set the tone” of the school, not only in that they put formal efforts into supporting teacher learning, but also in that they have a strong responsibility to meet the needs of teachers (Qian and Walker, 2020). This study further investigates how these leaders play their roles in the CoP of informal interactions. The TRG leaders and LPG leaders were regarded by the interviewees as the most frequent subjects for informal interactions for two possible reasons. First, group leaders are usually expert teachers within the school. This means that they have more teaching experience, and their achievements are relatively advanced. Therefore, their peers tend to ask them informally for help in addressing instructional problems. Second, group leaders are also the leaders in either the teaching research and/or lesson planning groups, which represent school-level management. This means they are the formal institutional managers of most other teachers, as they are in a position to announce school-level policies and tasks. They also may have more opportunities to participate in professional development activities at the district and national levels, which translates into more opportunities for communication with educational research experts, university researchers, and teachers in other districts. These “privileges” make each of the group leaders the “knowledgeable person” at school, which may encourage teachers to talk with them more frequently.

However, when comparing the two schools in this study, teachers at School A tended to interact with group leaders more often than those at School B. Teachers' relationships with group leaders could also be described as tenuous at both schools. This might be due to the “privileges” that group leaders have, as the privileges may also confer more authority, and this can strain relationships. In such cases, informal interactions between teachers can also be a channel for understanding, which may help solve any relational tensions (Shirrell et al., 2019). Research has shown the difficulties leaders may face at school (Adler and Alshwaikh, 2019; Widjaja and Vale, 2020). In this study, we also found that leaders must balance their dual roles to ensure that teachers maintain their willingness to interact informally with them.

### Influences on Teaching Practices From Teachers' Informal Interactions

As revealed through the interviews, teachers valued support and help from informal interactions with colleagues; this is in alignment with findings from previous research (e.g., Lewis and Lee, 2017). In reflecting on the effects and influences of informal interactions, teachers shared goals for and problems of cultivating the Domain of understanding principles and the standard, as well as improving teaching skills. Teachers thought they gained a clearer understanding of teaching requirements and the standard through informal interactions, and they said they knew how to adjust their strategies. This reflective process connects the shared goal of the Community with Practice (Wenger, 1998). It may allow teachers to generate value from informal interactions and further promote sustainable participation in informal interactions.

In Wenger's CoP framework, the Practice dimension results in an outcome level of participation in the Community (Siu, 2015), which suggests that teacher participation may encourage their reflections on teaching and their own roles. However, for teachers, less is known in the literature about what is reflected upon through informal interactions. Also, teachers in one educational context may reflect on different content than teachers in other subjects, levels of education, or even different countries and cultures. This study captures Chinese mathematics teachers' reflections on this only. For example, one participant said that after they received advice and suggestions from other colleagues through informal interactions, they tried it out in their teaching. Then, they might have had an extended conversation and discussion about whether this advice could be used in class, what to do if any problems occurred, and what the reasons might be for such problems. The analysis of this reflective assessment allows teachers to combine what they have gained from informal interactions with daily practice to improve their own teaching.

In addition to reflecting on teaching effects, some teachers also mentioned the role of broadening new horizons in the field, which echoes the findings from Sánchez (2011), in which teachers found it useful to communicate with other colleagues because they wanted to know what their peers thought of the same issue and what the differences and similarities might be. Even though reflections on informal interactions were captured in this study, questions have still arisen. For example, as cited in the literature, teachers' reflections on their own roles are crucial for developing intrinsic motivation for practice (Van Lankveld et al., 2016). However, in this study, it seemed that teacher self-reflections were uncommon. These are the main sources used to identify their roles in the community (Wenger, 1998), but because they do not regularly engage in the practice, we might not be able to fully understand how they form that community identity. One possible reason is cultural influence (e.g., Clarke et al., 2006); because of the conservative Asian culture, Chinese mathematics teachers may not be used to talking about themselves. Future research is needed to further investigate teachers' reflections on their own interactions with colleagues.

### The Mechanism of Informal Interactions Through the Lens of CoPs

It is not difficult to recognize the mutual connections among the three dimensions of Domain, Community, and Practice in the teacher interviews. Previous research has also noted that teachers' characteristics could be explained through the lens of CoP, but the mechanism by which these dimensions connect to each other has not been fully explored (e.g., Siu, 2015). We propose that a possible underlying process could be that the shared goals revealed from the dimension of Domain stimulate the need for informal interactions. The group leaders, either TRG or LPG, play their roles in encouraging teachers to initiate informal interactions, providing them with people to approach first, which shapes the community of informal interactions. We cannot separate the influences of informal interactions on the practice from the initial needs, which is why we found that teacher feedback in the dimension of Practice echoes what has been proposed in the dimension of Domain.

We propose that the recurring appearance of the three dimensions in informal interactions creates conditions for teachers to communicate with each other, which allows them to maintain their memberships in these groups. It has been noted that it is important for teachers to maintain memberships in groups, particularly in the strong cultures that exist within schools (Ponticell, 2003; Somekh, 2008). This study reveals that the dual roles of group leaders help shape the atmosphere of the groups and the schools; in this sense, teachers can determine whether they are encouraged to interact informally with these leaders and each other. Teachers then have the opportunity to benefit from informal interactions and build relationships within groups.

On the other hand, although this study provides evidence about the positive influences that arise from informal interactions between teachers, it is restricted to this study, and we are not able to conclude that informal interactions are always useful. As Zhao and Frank (2003) proposed, it could be difficult for teachers to make changes and bring educational innovations into practice where existing values and cultures are too strong. Therefore, even if teachers' informal interactions contribute to solving the problems at hand, the ways informal interactions can create sustained influence and further contribute to shaping a school's culture must still be investigated.

## Conclusion

This study uses the CoP framework to investigate Chinese mathematics teachers' informal interactions. A variety of informal interaction themes were identified in each dimension of the framework (Domain, Community, and Practice). The shared goals and need for sharing hands-on experiences among teachers form a foundation and create an interactive space for teachers to initiate informal interactions. Within the community, based on a Chinese school-based professional development background, the critical role of group leaders was recognized. The coherence between the shared Domain and the Practice was captured because teachers tend to realize their shared goal of informal interactions in practical teaching. The connections among the three dimensions also reveal how informal interactions are sustained and then influence their teaching practices. Overall, this study opened our eyes to understanding a specific form of professional development in daily teaching practice. Information gleaned from the interviews bolstered the opinion that teachers value their support networks and lean on each other to navigate the intricacies of their profession. These findings encourage educators to support building the CoP of informal interactions at the school level.

## Limitations

The limitations of this study should also be taken into consideration. First, because informal interactions happen spontaneously and casually in daily practice, it is difficult to include onsite observations or video recording analyses of the interactions themselves. More research methods and approaches must be developed to investigate similar situations at other schools. Additionally, although the nine interviews provided a holistic picture from each teacher's perspective, it would be difficult to say that their opinions represented those of all Chinese mathematics teachers. However, the purpose of this study was only to highlight a specific form of teacher collaboration in daily teaching and to help us understand a particular form of professional development. Also, although we conducted this research with mathematics teachers, we found that the teachers participating in informal interactions could reveal a possible common process of how teachers seek informal interactions at schools, with only differences in content. Further research about, for example, how school culture influences the shaping of the CoP of informal interactions at schools, and how specific content of informal interactions helps teachers learn and improve teaching, can be conducted. This would broaden the sample so teachers and other educators from different schools are included and various contexts are considered to help the research community better understand the nature of informal interactions and even possible cultural influences on them.

## Data Availability Statement

The data analyzed in this study is subject to the following licenses/restrictions: Dataset includes human being's pictures and voices, so it was not publicly available. Requests to access these datasets should be directed to shu.zhang@bnu.edu.cn.

## Author Contributions

All authors contributed to the writing of this paper. SZ and WZ were in charge of the study conception and design. SZ and YC was involved in the collection and analysis of the Chinese data. SZ and WZ was involved in the analysis of the data. The first draft of the manuscript was written by SZ and all authors commented on previous versions of the manuscript. All authors read and approved the final manuscript.

## Conflict of Interest

The authors declare that the research was conducted in the absence of any commercial or financial relationships that could be construed as a potential conflict of interest.

## Publisher's Note

All claims expressed in this article are solely those of the authors and do not necessarily represent those of their affiliated organizations, or those of the publisher, the editors and the reviewers. Any product that may be evaluated in this article, or claim that may be made by its manufacturer, is not guaranteed or endorsed by the publisher.
